# Clinical outcomes of anterior prostate cancers treated with robotic assisted radical prostatectomy

**DOI:** 10.1002/bco2.202

**Published:** 2022-10-27

**Authors:** Reyan Saghir, Beth Russell, Francesca Kum, Raef Darwish, Jude Deane, Christopher Allen, Hira Rizwi, Noman Saghir, Nikhil Mayor, Paul Cathcart, Prokar Dasgupta, Rick Popert, Christian Brown, Ben Challacombe

**Affiliations:** ^1^ Guy's Hospital London UK; ^2^ Cancer Epidemiologist Research Associate King's College London London UK; ^3^ King's College London London UK; ^4^ University Hospital of North Durham Durham UK

**Keywords:** anterior, continence, erectile function, prostate cancer, PSA, radical prostatectomy, RARP, roboticassisted

## Abstract

**Introduction:**

A prospective cohort study comparing peri‐ and postoperative outcomes for patients with predominantly anterior prostate cancer (APC) identified preoperatively against non‐anterior prostate cancer (NAPC) treated via robotic‐assisted radical prostatectomy (RARP).

**Patients and Methods:**

Of the 757 RARP's completed between January 2016 and April 2018, two comparative cohorts for anterior and an equivalent group of non‐anterior prostate tumours each consisting of 152 patients were compared against each other. Data were collected on the following variables: patient age; operating consultant; preoperative PSA, ISUP grade, degree of nerve sparing; tumour staging; presence and location of positive surgical margins; PSA density, postoperative ISUP grade; treatment paradigm and postoperative PSA, erectile function, and continence outcomes with 2‐year follow‐up.

**Results:**

APCs were found to have significantly lower ISUP grading postoperatively; increased diagnosis via active surveillance over new diagnosis; more frequently undertaken bilateral nerve‐sparing and long‐term poorer continence outcomes at 18 and 24 months postoperatively (*p* < 0.05). Pre‐ and post‐op PSA levels, erectile function, PSA density, positive surgical margins (PSM), age and tumour staging showed no significant differences between the APC and NAPC cohorts (*p* > 0.05).

**Conclusion:**

The lower ISUP grading could indicate APC as overall being less aggressive than NAPC, whereas the poorer long‐term continence outcomes require further investigating. The non‐significant differences amongst tumour staging, PSA density, preoperative PSA levels and PSM rates suggest that APC may not be as significant as predicted in diagnostic evaluation. Overall, this study provides useful information on the growing literature of anterior prostate cancer. Being the largest comparative cohort study to date on APC post‐RARP, these results indicate the true characteristics of anterior tumours and their functional outcomes to help improve education, patient expectations and management.

## 1 INTRODUCTION

Robotic‐assisted radical prostatectomy (RARP) is currently the gold standard surgical treatment for localised prostate cancer.^1^ Development of the da Vinci (Intuitive®) surgical system and preliminary trialling of its application in a urological setting led to the first documented RARP occurring in early 2000.^1^, ^2^, ^3^ Following this, the use of the da Vinci system has been adopted in many centres worldwide. Guy's and St Thomas' Hospitals were one of the first to pioneer the use of robotic assistance in the urological setting in the United Kingdom in 2004 with over 5000 cases performed.

With a greater understanding of utilising robotics to treat prostate cancer, we are constantly trying to improve surgical practice achieve better patient outcomes. As such prostate specific antigen (PSA) levels, continence and erectile function are still concerning to patients' disease‐free survival and quality of life.^4^, ^5^ Although recent studies have documented various improvements in postoperative care, management, and surgical technique, preoperatively identifying individuals at a higher risk of a poorer prognosis is also very important. This study was therefore performed to observe the effect location of prostate cancer identified preoperatively has on the subsequent outcomes mentioned above peri‐ and postoperatively.

Understanding of anterior prostate cancers is a developing field in urology with a growing base of knowledge. Typically, when patients present to their General Practitioner and a digital rectal exam (DRE) is performed for an anterior prostate cancer patient, this may be ‘normal’ as only the posterior aspect can be felt. As a result, these anterior cancers may be missed or diagnosed later when more extensive throughout the prostate on repeated MRI imaging or when the patient becomes symptomatic. It is estimated that anterior prostate cancers account for approximately between 13% and 30% of all cases.^6^, ^7^, ^8^, ^9^ As such, many hypothesise that as these cancers may be late presenting due to being potentially missed on initial investigation, they would have poorer postoperative outcomes following RARP, especially regarding biochemical recurrence, positive surgical margin rate, continence, and erectile function.

As part of this study, we want to identify the differences between predominantly anterior and non‐anterior prostate cancer patient cohorts identified preoperatively and their subsequent peri‐ and postoperative outcomes, to improve education and patient expectations and management.

## 2 PATIENTS AND METHODS

This study is a prospective comparative cohort analysis of all RARPs performed at a high‐volume tertiary robotic centre between January 2016 and April 2018. During this period, a total of 757 RARP procedures were performed.

**FIGURE 1:**
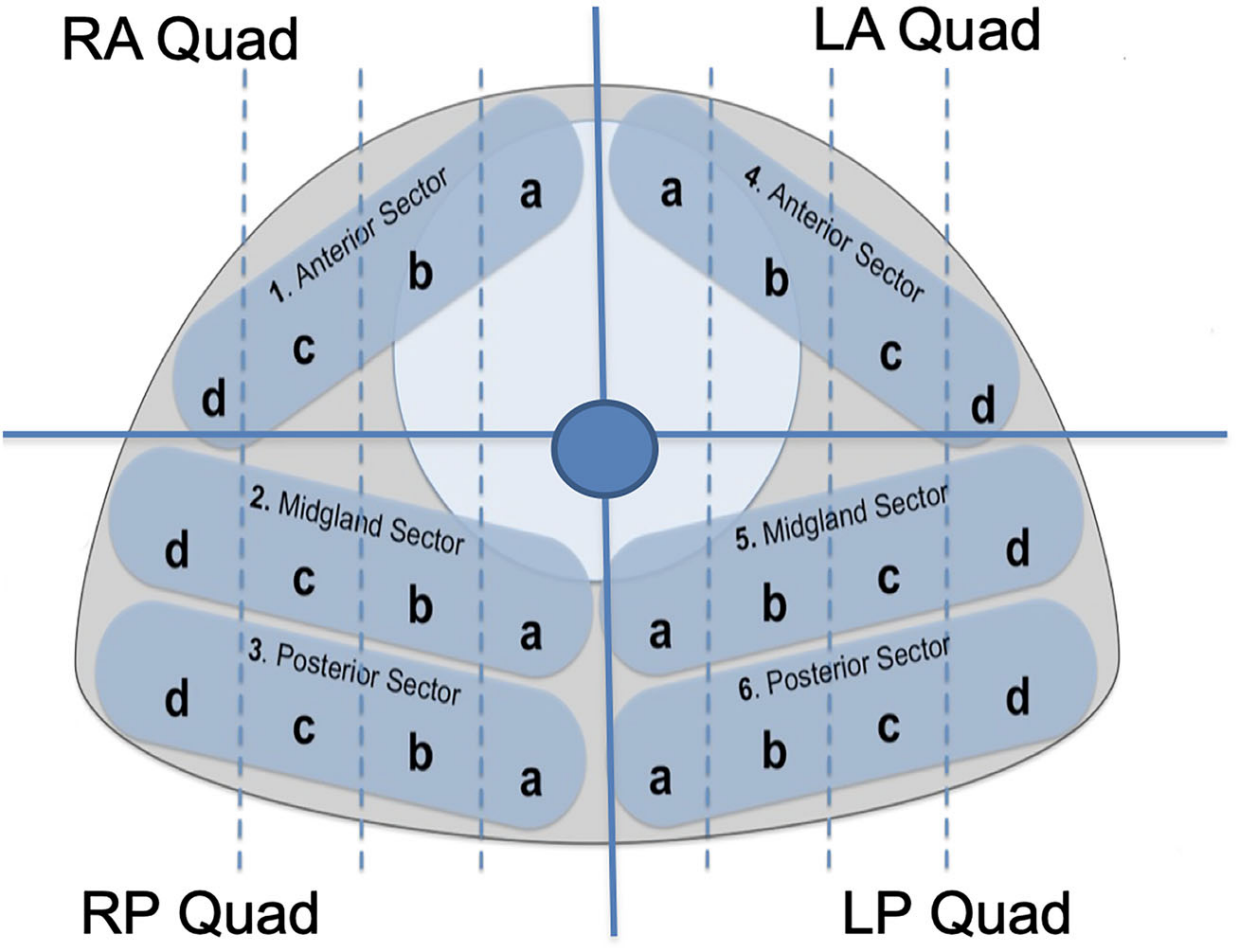
Cross‐sectional diagram of the prostate gland (coronal plane) delineated into individual sectors and quadrants correlating to location of tumour on biopsy and MRI. RA, right anterior; LA, left anterior; RP, right posterior; LP, left posterior

From this group of patients, we defined our anterior prostate cancer group as those whom we intended to treat as having anterior prostate cancer. Therefore, individuals in which predominantly the preoperative biopsy cores were anterior rather than posterior or if on multiparametric MRI imaging anterior cancer was identified as most prevalent these patients formed our ‘anterior prostate cancer’ group.^10^, ^11^, ^12^ Figure 1 displays a template diagram, which delineates the boundaries used for defining anterior and posterior prostate cancers on both biopsy and MRI.

Of the 757 RARP's performed 210 patients (27.7%) were found to have predominantly anterior prostate cancer identified preoperatively either on biopsy or MRI and as such 547 were non‐anterior (72.3%). Evaluating the anterior prostate cancer cohort, we removed any patients for whom biopsy was performed transrectally (TRUS) rather transperineally, as multiple studies have shown that TRUS is inferior and inadequate for tumours in this position, with the transperineal (TP) approach preferred.^6^, ^13^ Also, patients were removed for whom complete data for 2 years follow‐up was insufficient regarding their postoperative outcomes. This led to a total cohort number of 152 anterior prostate cancer patients for analysis. We then, using a random number generator, identified a secondary 152 non‐anterior prostate cancer cohort to compare against. Similar to the anterior cohort, any patients with missing data or who underwent transrectal biopsy were removed.

Each patient received a standard preoperative workup: PSA, pre biopsy bi‐parametric MRI and appropriate prostate biopsies taken within 14 days of first referral. All patients were discussed at the Multi‐Disciplinary Team (MDT) meeting in which tumour grade, stage and lymph node dissection was decided, with a management plan agreed and as per the network target by day 62 from initial presentation to the first definitive treatment.^14^

All operative procedures were carried out according to local trust protocol. Patients received prophylactic gentamycin and intravenous co‐amoxiclav at induction. Patients were positioned in the Trendelenburg position with end docking and standard 6 port access for the dual console Da Vinci Xi or Si (Intuitive®) Surgical System. Postoperatively, all patients were mobilised on the first postoperative day with their urethral catheter in situ and discharged with low molecular weight heparin injections for 28 days. Patients then had a nurse‐led trial without catheter at week 1 and surgeon review at 8 weeks post‐RARP.

Data were collected on 11 perioperative variables and were correlated to three long‐term postoperative outcomes, also referred to as the trifecta: PSA levels at 6 weeks, 12 months, and 24 months; urinary continence and ED at 6, 12, 18 and 24 months. An undetectable PSA was deemed as <0.1 μg/L. Continence endpoints were defined by individuals either fully continent or using a security pad for light leakage followed by increased number of pad usage per day. The endpoint for complete ED was ED refractory to oral agent, pump, or injections. The perioperative variables examined included patient age; operating consultant; preoperative PSA, ISUP grade, amount of nerve sparing (none, unilateral or bilateral); tumour staging; presence and location of positive surgical margins; PSA density, postoperative ISUP grade and treatment paradigm (newly diagnosed or active surveillance).

### 2.1 Statistical analyses

Descriptive statistics were used to describe the demographic and clinical characteristics of the patients based on location of tumour (i.e., anterior vs. non‐anterior). For all such perioperative variable data collected, appropriate Kruskal–Wallis, *χ* ^2^ and *t*‐test statistical analyses were performed between both anterior and non‐anterior tumour groups.

Logistic regression analyses were then applied to estimate odds ratios (ORs) as a measure of association between tumour location and relative risk of: being continent at 6, 12 and 18 months (defined as fully continent or use of security pad), having a detectable post‐op PSA (≥0.03) at 6 weeks, 12 and 24 months, positive operative margins and erectile dysfunction at 6, 12, 18 and 24 months (defined as managed well with oral agents and pump, managed well with injections, trial period of other oral agents and pump, not managed well with oral agent or pump, not managed well with oral agents, pump and injections or trial injections). Patients who did not wish to pursue treatment were excluded when considering erectile function. All analyses were adjusted for age at surgery.

This study has been registered as an audit at our institution (Ref No: 8077), and all work has been reported in line with the STROCSS criteria.^15^ No ethical approval or funding was required for this study.

## 3 RESULTS

A total of 757 RARPs were carried out between January 2016 and April 2018 (mean 31/month), of which two cohorts were formed for comparative analysis each comprising 152 RARP's identified as either anterior or non‐anterior preoperatively. The following tumour characteristic and patient demographic is displayed in Table 1.

**TABLE 1:** Tumour and patient characteristics stratified by tumour location (anterior vs. non‐anterior)

Variable	Total (*N* = 304)		Anterior (*N* = 152)		Non‐anterior (*N* = 152)		*p*‐value
	n	%	n	%	n	%	
Age groups							0.512
<50	22	7.20	14	9.20	8	5.30	
50–59	98	32.20	50	32.90	48	31.60	
60–69	149	49.00	68	44.70	81	53.30	
70–79	34	11.20	19	12.50	15	9.90	
80+	1	0.30	1	0.70	0	0.00	
Consultant							0.002
Operating Consultant A	39	12.80	14	9.20	25	16.40	
Operating Consultant B	54	17.80	33	21.70	21	13.80	
Operating Consultant C	20	6.60	10	6.60	10	6.60	
Operating Consultant D	134	44.10	77	50.70	57	37.50	
Operating Consultant E	57	18.80	18	11.80	39	25.70	
Preoperative PSA							0.484
<1	2	0.70	1	0.70	1	0.70	
1–4	57	18.80	29	19.10	28	18.40	
5–9	138	45.40	66	43.40	72	47.40	
10–14	59	19.40	36	23.70	23	15.10	
15–19	44	14.50	20	13.20	24	15.80	
Missing	4	1.30	0	0.00	4	2.60	
Preoperative Gleason score							0.469
ISUP 1	25	8.20	14	9.20	11	7.20	
ISUP 2	163	53.60	83	54.60	80	52.60	
ISUP 3	74	24.30	30	19.70	44	28.90	
ISUP 4	15	4.90	6	3.90	9	5.90	
ISUP 5	17	5.60	9	5.90	8	5.30	
Missing	10	3.30	10	6.60	0	0.00	
Treatment paradigm							0.017
New diagnosis	170	55.90	75	49.30	95	62.50	
Active surveillance	132	43.40	77	50.70	55	36.20	
Missing	2	0.70	0	0.00	2	1.30	
Surgical margins							0.094
Negative	241	79.30	113	74.30	128	84.20	
Positive <3 mm	45	14.80	29	19.10	16	10.50	
Positive >3 mm	15	4.90	7	4.60	8	5.30	
Missing	3	1.00	3	2.00	0	0.00	
Positive margin location							0.220
None	248	81.60	117	77.00	131	86.20	
Apical	24	7.90	15	9.90	9	5.90	
Circumferential	19	6.30	12	7.90	7	4.60	
Base	13	4.30	8	5.30	5	3.30	
Nerve sparing							0.018
None	38	12.50	20	13.20	18	11.80	
Unilateral	116	38.20	46	30.30	70	46.10	
Bilateral	149	49.00	85	55.90	64	42.10	
Missing	1	0.30	1	0.70	0	0.00	
T staging							0.386
T2	209	68.80	101	66.40	108	71.10	
T3a	62	20.40	34	22.40	28	18.40	
T3b	30	9.90	17	11.20	13	8.60	
T3b N1	3	1.00	0	0.00	3	2.00	
PSA density							0.235
<0.10	35	11.50	17	11.20	18	11.80	
0.10–0.14	60	19.70	32	21.10	28	18.40	
0.15–0.25	97	31.90	41	27.00	56	36.80	
25+	103	33.90	58	38.20	45	29.60	
Missing	9	3.00	4	2.60	5	3.30	
Postoperative Gleason score							0.019
ISUP 1	9	3.00	5	3.30	4	2.60	
ISUP 2	209	68.80	113	74.30	96	63.20	
ISUP 3	53	17.40	20	13.20	33	21.70	
ISUP 4	13	4.30	8	5.30	5	3.30	
ISUP 5	16	5.30	3	2.00	13	8.60	
Missing	4	1.30	3	2.00	1	0.70	

The modal patient age group was between 60 and 69 years with the anterior group age being 61.4 ± 6.44 and non‐anterior 60.7 ± 7.62 years. Mean preoperative PSA of both groups combined was 10.1 ng/ml with the anterior group being 10.2 ± 8.17 ng/ml and non‐anterior 10.03 ± 7.64 ng/ml. Both age and preoperative PSA levels were not statistically different between the groups (*p* > 0.05). Differences in number of anterior and non‐anterior cases performed by each operating surgeon however were statistically significant (*χ* ^2^ = 16.5, *p* = 0.02), and this was adjusted for in multivariate analysis.

The modal preoperative ISUP grade was 2 amongst both groups followed by an ISUP grade 4, grade 1, grade 3 and lastly grade 5, and this distribution was non‐statistically significant between both groups on preoperative analysis formed via biopsy and MRI (*χ* ^2^ = 2.53, *p* = 0.469). However postoperatively on removal of the prostate specimen although the modal distribution was still the same a statistically significant difference was noted whereby a higher proportion of non‐anterior cancers were identified as having a higher ISUP grade compared with the anterior group (*χ* ^2^ = 11.7, *p* = 0.019). Also interestingly comparing the preoperative ISUP grades with the postoperative ISUP grades attained for both anterior and non‐anterior groups, a statistically significant difference was noted (*χ* ^2^ = 36.39, *p* < 0.001 and *χ* ^2^ = 67.5, *p* < 0.001, respectively).

Within the anterior group, 49.3% of patients were newly diagnosed cancers on first presentation compared with 62.5% in the non‐anterior group. This difference between the groups was identified as being statistically significant (*χ* ^2^ = 4.42, *p* = 0.017).

No statistically significant difference was noted in the positive surgical margin rate of the anterior group 25.7% compared with 15.8% in the non‐anterior group (*χ* ^2^ = 4.72, *p* = 0.094). Similarly, PSA density and tumour staging also showed no statistically significant difference between the groups (*χ* ^2^ = 4.25, *p* = 0.235 and *χ* ^2^ = 0.750, *p* = 0.386, respectively). The location of positive margins identified was most prevalent in the apical region, followed by circumferentially then in the bases of the prostate gland for both anterior and non‐anterior tumours (*χ* ^2^ = 4.42, *p* = 0.22).

Bilateral or unilateral nerve sparing occurred in 88.2% of the non‐anterior cancer cohort compared with 86.8% in the anterior cancer group. However, of this percentage, 55.9% of the anterior group had bilateral nerve sparing compared with 42.1% of the non‐anterior group. This difference was statistically significant (*χ* ^2^ = 8.03, *p* = 0.018).

Table 2 displays the logistic regression analyses performed on the following postoperative outcomes assessed; PSA levels at 6 weeks, 12 months, and 24 months; continence and erectile function outcomes at 6, 12, 18 and 24 months postoperatively and positive surgical margin rates between both anterior and non‐anterior prostate cancer groups. Of these analyses, the only relationship in which a statistically significant difference was noted was a difference in continence outcomes with the anterior cohort having poorer continence outcomes at 18 and 24 months postoperative compared with the non‐anterior group (*p* < 0.05).

**TABLE 2:** Logistic regression analyses for postoperative outcomes; PSA level, continence, erectile function, and positive surgical margin rate stratified by tumour location (anterior vs. non‐anterior)

Variable	*n*/Total anterior or non‐anterior	OR (unadjusted)	95% CI	OR (age adjusted)	95% CI
**Continent @ 6 months**					
Non‐anterior	115/150	1.00	Ref.	1.00	Ref.
Anterior	103/144	0.76	(0.45–1.29)	0.78	(0.46–1.32)
**Continent @ 12 months**					
Non‐anterior	124/144	1.00	Ref.	1.00	Ref.
Anterior	108/139	0.56	(0.30–1.04)	0.58	(031–1.09)
**Continent @ 18 months**					
Non‐anterior	127/143	1.00	Ref.	1.00	Ref.
Anterior	110/139	0.48	(0.25–0.93)	0.49	(0.25–0.96)
**Continent @ 24 months**					
Non‐anterior	129/141	1.00	Ref.	1.00	Ref.
Anterior	110/139	0.35	(0.17–0.72)	0.36	(0.18–0.75)
**Detectable postoperative PSA @ 6 weeks**					
Non‐anterior	27/152	1.00	Ref.	1.00	Ref.
Anterior	19/151	0.67	(0.35–1.26)	0.65	(0.34–1.23)
**Detectable postoperative PSA @ 12 months**					
Non‐anterior	19/149	1.00	Ref.	1.00	Ref.
Anterior	26/148	1.46	(0.77–2.77)	1.41	(0.74–2.70)
**Detectable postoperative PSA @ 24 months**					
Non‐anterior	22/146	1.00	Ref.	1.00	Ref.
Anterior	23/136	1.15	(0.61–2.17)	1.13	(0.60–2.15)
**Positive margins**					
Non‐anterior	24/152	1.00	Ref.	1.00	Ref.
Anterior	36/149	1.70	(0.96–3.02)	1.69	(0.94–3.01)
**Erectile dysfunction @ 6 months**					
Non‐anterior	124/138	1.00	Ref.	1.00	Ref.
Anterior	117/134	0.78	(0.37–1.65)	0.80	(0.37–1.70)
**Erectile dysfunction @ 12 months**					
Non‐anterior	107/123	1.00	Ref.	1.00	Ref.
Anterior	107/125	0.89	(0.43–1.83)	0.91	(0.44–1.87)
**Erectile dysfunction @ 18 months**					
Non‐anterior	91/114	1.00	Ref.	1.00	Ref.
Anterior	102/125	1.12	(0.59–2.13)	1.12	(0.59–2.14)
**Erectile dysfunction @ 24 months**					
Non‐anterior	83/110	1.00	Ref.	1.00	Ref.
Anterior	101/125	1.37	(0.74–2.55)	1.37	(0.74–2.55)
**Upgrade of ISUP grade**					
Non‐anterior	31/151	1.00	Ref.	1.00	Ref.
Anterior	18/141	0.56	(0.30–1.06)	0.56	(0.30–1.06)

## 4 DISCUSSION

This study is the largest prospective cohort analysis performed to date assessing the pathological and clinical features of anterior prostate cancers, peri‐ and postoperatively. Not only in the number of cases of anterior prostate cancer analysed prospectively but also in the depth of variables.

Assessing the demographic and clinical characteristic data, statistically significant differences were identified within the perioperative data collected. First is the change in postoperative ISUP grades. With the postoperative ISUP grades providing the truest reflection of the now resected cancer and can be fully analysed, our results showed a significant difference between the groups with a greater proportion of the non‐anterior group having higher ISUP graded cancers compared with the anterior group. From this we can infer that on a histopathological level anterior cancers may be less severe than non‐anterior cancers. Similar such results whereby lower Gleason scores amongst anterior prostate cancers have also been reported by Koppie et al. and Magers et al., whereas Mastumoto et al. noted anterior prostate tumours as less likely to invade extraprostatic tissues.^16^, ^17^, ^18^

Also, on a different note, our study has highlighted a significant difference in the ISUP grades assigned preoperatively compared with postoperatively for both anterior and non‐anterior prostate cancers. Our protocol used transperineal biopsy for all patients in which a systematic approach would most often be adopted alongside targeted sampling if indicated previously, for example, via preoperative MRI imaging. However, the fact that we have noticed consistent upgrading from pre‐ to postoperative ISUP scores, it may suggest some inadequacies in this sampling technique despite its perceived thoroughness.

Another statistically significant difference highlighted by our results showed that anterior cancers were identified more frequently via active surveillance rather than as a primary diagnosis, as seen in the non‐anterior cohort. The reasoning behind this difference can be explained as pre‐2016 many of these patients who were undergoing active surveillance would have received a transrectal ultrasound biopsy (TRUS). As such, as TRUS biopsies cannot easily sample the anterior region, their initial sampling result would have been negative for an anterior predominant prostate cancer; later, however, with change in technique to transperineal, the anterior region was sampled more effectively and thus led to a higher diagnostic rate amongst this population. Anterior tumours may also be more challenging to initially identify in the anterior stromal area or develop more insidiously over time.

The degree of nerve sparing was also a significant difference noticed between the anterior and non‐anterior cohorts. For anterior prostate cancers an increased recommendation for bilateral nerve sparing rather than unilateral nerve sparing was observed. The decision to nerve spare is generated during the preoperative surgical planning meeting, however the degree of unilateral or bilateral sparing is at the discretion of individual surgeons. A possible explanation could be related to the anatomical positioning of the prostatic neurovascular bundles (NVB). Residing posterolaterally, when considering a predominantly anterior cancer these posterior structures are not at risk of local invasion, as such, a wide margin excision is not needed in the posterior aspect which means the NVB has a higher chance of being spared. This theory is further evidenced whereby an expected rise in positive surgical margins would be expected with increased bilateral nerve sparing procedures, as a narrower excision has been performed to keep the NVB intact. However, as the positive surgical margin rate showed no significant difference between both anterior and non‐anterior cohorts this demonstrates that cancer is not being left behind at the expense of bilateral nerve sparing in RARP for anterior prostate cancer.

Our results also are useful in providing new information to the literature on anterior prostate cancers through the non‐statistically significant differences between the variables as well. From our experience, we were expecting the data to show a trend towards anterior prostate cancers being much more aggressive and locally‐advanced, due to DRE being unable to feel the prostates anterior surface^7^ and the perceived volume of tumour on preoperative MRI. However, our results found no statistically significant differences amongst preoperative PSA levels, like Mygatt et al., tumour staging or PSA density (*p* > 0.05),^19^ and in fact, the ISUP grading on the postoperative specimen was lower in the anterior group than non‐anterior. Our results are different to previous smaller scale published studies such as by Mygatt et al., Koppie et al. and Falzarano et al. who stated a higher positive surgical margin amongst anterior prostate cancers patients,^7^, ^17^, ^19^ but these studies may not have had the surgery guided by pre‐biopsy MRI staging as in ours.

Assessing the postoperative outcomes using logistic regression analysis, again the lack of significant differences between the anterior and non‐anterior cohorts provides useful new information to the literature, previously unreported. Observing PSA levels for biochemical recurrence at 6 weeks, 12 months, and 24 months, no significant difference was found; similarly, as mentioned above, no differences in positive surgical margin rate was found. These findings support similar results published by Falzarano et al. documenting no differences in postoperative PSA levels found in 5‐year follow‐up.^7^ This means from a managing patient expectation perspective for an individual with anterior prostate cancer, their odds ratio of recurrence is the same as non‐anterior, and similarly, there is no greater or lesser risk of a positive margin. Likewise, our analyses also showed no significant difference in erectile function outcomes postoperatively at 6, 12, 18 and 24 months postoperatively. As such, this is useful for the urologist to be aware of as although increased bilateral nerve sparing was present in our anterior cohort, this did not relate to significantly improved erectile function in the long‐term comparative to RARP's performed for non‐anterior prostate cancer patients.

Continence outcomes however did show a significant difference in the long term at months 18 and 24 whereby our anterior cohort had significantly poorer continence outcomes needing more than one security pad compared with the non‐anterior group (91% vs. 79% at 24 months). This is a new finding to the literature and a possible explanation could be related to the degree of wide excision for apical anterior prostate cancers with an inability to perform a close apical dissection and maximal urethral preservation in many cases. As previously reported, although in our study, the positive surgical margin rate was non‐significant and acceptable at 23.7%, others have stated rates as high as 40% in these tumours, as dissection may lead to wider excisions when planning to operate. This in turn likely involves shortening of the urethral stump at the anastomosis due to a compromised apical dissection thus taking either longer to functionally recover or having a permanently worse outcome overall.^16^, ^17^

## 5 LIMITATIONS

For this study five highly experienced urologists (>500 cases each) performed all RARP procedures in conjunction with dedicated robotic fellows; however, intra‐operator variability could be present within the results especially regarding outcomes such as positive surgical margins and postoperative continence outcomes.^20^ Previous literature concerning anterior prostate cancers have stated race as being unequivocal, whereas others such as Sato et al. feel a racial propensity for instance amongst the Japanese population may exist with anterior prostate cancer, race was a variable not adjusted for in our data collection^19^, ^21^ although 50% of our RARPS generally are on Afro‐Caribbean men. Other limitations include our methodology; we classified anterior prostate cancers based on preoperative assessment as those we intended to treat as anterior cancers based on the transperineal biopsy and MRI results. However, prior smaller scale studies into anterior prostate cancer have shown that firstly targeted biopsy can yield a higher diagnostic rate for anterior cancer, and this was not performed for every patient as a systematic approach was adopted with operator choice for targeting.^9^, ^22^ Also, studies have shown that reporting radiologist education having identified the differences between anterior and non‐anterior prostate cancers can have a significant difference in the number of anterior cancers reported, this was not considered.^23^ Also, by collecting data on so many variables over a 2‐year follow up period, missing data are present for a very small proportion, which could influence the results as well.

## 6 CONCLUSION

Overall, this prospective comparative cohort study provides an extensive fundamental basis to understanding both the perioperative and postoperative outcomes for anterior prostate cancers in relation to the more well reported non‐anterior cancers. The study has identified new significant differences whereby anterior cancers have a lower postoperative ISUP grade, increasing diagnosis via active surveillance, increased bilateral nerve sparing opportunities and poorer long‐term continence outcomes compared with the non‐anterior cohort. Meanwhile, the non‐significant differences in pre‐ and postoperative PSA scores, tumour staging, positive surgical margins, PSA density and erectile function can help guide patient management and expectations.

## CONFLICTS OF INTEREST

None declared.

## AUTHOR CONTRIBUTIONS

RS was the lead author involved with the data collection, analysis and article synthesis with significant support and conception of idea from supervising author BC. Statistical analysis was performed by BR. FK, NM and NS provided significant support in manuscript development. RD, JD, CA and HR supported in data collection, and data were provided from fellow consultant urologists PC, PD, RP, CB and BC.
